# Complete genomes of 12 *Streptococcus salivarius* strains and one *Streptococcus raffinosi* strain isolated from the human oral cavity and gastric lavage

**DOI:** 10.1128/mra.00228-26

**Published:** 2026-07-13

**Authors:** Merlin Brychcy, Melissa Mildenberger, W. Florian Fricke, Nina S. Schmidt, Christian Sina, Herbert Schmidt

**Affiliations:** 1 Department of Food Microbiology and Hygiene, University of Hohenheim 26558, Stuttgart, Germany; 2 Department of Microbiome Research and Applied Bioinformatics, University of Hohenheim 26558, Stuttgart, Germany; 3 Institute of Nutritional Medicine, University of Lübeck and University Medical Center Schleswig-Holstein 666505, Lübeck, Germany; Loyola University Chicago, Chicago, Illinois, USA

**Keywords:** *Streptococcus salivarius*, oral microbiome, whole genome, saliva

## Abstract

In this study, 11 *Streptococcus salivarius* strains, one *Streptococcus raffinosi* strain isolated from the human oral cavity and a gastric lavage sample, as well as one *S. salivarius* reference strain, were whole-genome sequenced to investigate their genomic heterogeneity. Bioinformatic analysis indicated two major clades.

## ANNOUNCEMENT

*Streptococcus salivarius* is a commensal inhabitant of the oral cavity and phylogenetically closely related to *Streptococcus thermophilus* (1, 2). The bacterium has been determined as both a possible pathogen (3) and a potential probiotic (4, 5), depending on the strain. However, comprehensive, comparative genomic analysis of this species is still limited. With this study, we aim to shed light on the diversity of the species by sequencing 11 *S. salivarius*, one *Streptococcus raffinosi* isolated from saliva and a gastric lavage, and one *S*. *salivarius* reference strain.

To isolate *S. salivarius* from the previously described sources (6), the samples were serially diluted from 10^−1^ to 10^−5^ and spread-plated in duplicate on tryptic soy agar (TSA, Becton Dickinson) with 0.3% (wt/vol) yeast extract and on Mitis-Salivarius agar (Sigma-Aldrich) with 1% (wt/vol) tellurite. Plates were incubated aerobically at 37°C for 24–48 h. Putative *S. salivarius* colonies were identified by their bluish “gum-drop” morphology, purified by single-colony streaking, and confirmed by 16S rRNA gene sequencing (Table 1).

**TABLE 1:** Strain information, sequencing quality metrics, and accession numbers of the strains investigated in this study

Strain designation^*a*^ and BioSample number	Internal reference	Isolation source	Geographical location	Genome length (bp)	Chromosome length (bp)	Plasmid lengths (bp) and topology	GC content (%)	Total reads Illumina	Total reads PacBio	N50 PacBio	Coverage	Number of coding genes	Number of coding sequences	GenBank and SRA accession	16S rRNA accession
DSM 20560T SAMN46560512	LTH 2477	Blood	Unknown	2,247,081	2,247,081	/^*b*^	40	3,867,193	43,923	9,237	150	2,131	2,041	CP180474 SRR32255291 SRR32255292	PQ683980
M14 SAMN46560513	LTH 7176	Gastric lavage	UKSH Campus Lübeck, Germany	2,417,055	2,274,530	142,525, circular	40	4,108,520	40,253	8,282	185	2,357	2,267	CP180472–CP180473 SRR32255269 SRR32255280	PQ683982
106-3 (CLE3) SAMN46560515	LTH 7254	Saliva	UKSH Campus Lübeck, Germany	2,311,765	2,159,556	152,209, circular	40	3,298,807	41,134	9,777	155	2,142	2,052	CP180468–CP180469 SRR32255265 SRR32255266	PQ683989
115-1 SAMN46560516	LTH 7284	Saliva	UKSH Campus Lübeck, Germany	2,255,301	2,255,301	/	40	5,417,335	51,775	8,204	150	2,136	2,046	CP180467 SRR32255263 SRR32255264	PQ683996
116-2 SAMN46560517	LTH 7291	Saliva	UKSH Campus Lübeck, Germany	2,469,665	2,245,303	133,724, circular; 76,090, linear; 14,548, linear	40	6,007,990	65,082	8,617	205	2,357	2,267	CP180463–CP180466 SRR32255289 SRR32255290	PQ684003
168-1 SAMN46560518	LTH 7293	Saliva	UKSH Campus Lübeck, Germany	2,161,892	2,161,892	/	40	5,645,925	66,540	9,092	220	2,031	1,941	CP180462 SRR32255287 SRR32255288	PQ684005
184-1 SAMN46560519	LTH 7299	Saliva	UKSH Campus Lübeck, Germany	2,287,090	2,287,090	/	40	5,693,654	45,163	8,090	130	2,184	2,094	CP180461 SRR32255285 SRR32255286	PQ684009
233-1 SAMN46560525	LTH 7305	Saliva	UKSH Campus Lübeck, Germany	2,105,750	2,105,750	/	40	5,644,718	130,657	3,726	200	1,992	1,902	CP180451 SRR32255272 SRR32255273	PQ684013
213-2 SAMN46560520	LTH 7309	Saliva	UKSH Campus Lübeck, Germany	2,194,710	2,194,710	/	40	5,410,032	74,992	5,848	140	2,069	1,979	CP180460 SRR32255283 SRR32255284	PQ684016
239-1 SAMN46560521	LTH 7310	Saliva	UKSH Campus Lübeck, Germany	2,206,796	2,206,796	/	40	5,353,055	52,299	6,607	100	2,091	2,001	CP180459 SRR32255281 SRR32255282	PQ684017
GP06-1 SAMN46560522	LTH 7312	Saliva	UKSH Campus Lübeck, Germany	2,483,258	2,281,989	167,217, circular; 34,052, linear	40	5,047,329	67,103	8,336	175	2,396	2,305	CP180456–CP180458 SRR32255278 SRR32255279	PQ684018
GP09-1 SAMN46560523	LTH 7319	Saliva	UKSH Campus Lübeck, Germany	2,564,378	2,359,717	204,661, circular	40	4,690,053	82,069	7,469	190	2,492	2,402	CP180454–CP180455 SRR32255276 SRR32255277	PQ684022
GP09-2 SAMN46560524	LTH 7320	Saliva	UKSH Campus Lübeck, Germany	2,408,912	2,155,673	253,239, linear	40	5,008,104	120,063	4,834	210	2,271	2,181	CP180452–CP180453 SRR32255274 SRR32255275	PQ684023

^*a*^ Strain 20560T originates from a public strain collection (DSMZ, Braunschweig, Germany). All other strains originate from samples derived from a clinical study (6, 7) (with approval by the ethics committee of the University Hospital Schleswig-Holstein, Germany).

^*b*^ /, no plasmids detected.

Genomic DNA was isolated after incubation of a single colony in TS broth at 37°C for 24 h using the Qiagen Blood and Tissue Kit (Hilden, Germany) according to the manufacturer’s protocol for gram-positive bacteria and assessed for quality using a NanoDrop device and agarose gel electrophoresis.

Genome sequencing and assembly were performed by Biomarker Technologies (BMK) GmbH (Münster, Germany) as previously reported (8). Briefly, a hybrid genome assembly and sequencing approach was used to generate a high-coverage genome assembly by combining Pacific Biosciences (PacBio) Revio long-read sequencing to generate a draft genome, with Illumina NovaSeq X Plus for further refinement. For Illumina sequencing, libraries were prepared by enzymatic fragmentation and PCR amplification using the VAHTS Universal Plus DNA Library Prep Kit (Vazyme, China). Subsequently, products were purified using VAHTS DNA Clean Beads (Vazyme, China). A NovaSeq X Plus with 150 bp paired-end reads was used for library sequencing. Read quality control was performed with fastp (9) using parameters “-q 10 -u 50 -y -g -Y 10 -e 20 -L 100 -b 150 -B 150,” fastuniq (10), and Trimmomatic (11) with “LEADING:3 TRAILING:3 SLIDINGWINDOW:50:20 MINLEN:10.” For PacBio sequencing, genomic DNA was sheared using a g-TUBE (Covaris, USA) and subsequently subjected to nick repair and end repair. SMRTbell adapters (using SMRTbell Prep Kit 3.0) were ligated to the repaired fragments, and the products were treated with exonuclease. Size selection of fragments was performed using BluePippin (Sage Science, Beverly, MA, USA). Primer and polymerase were bound to the library using the Revio Polymerase Kit (PacBio, USA), and complexes were purified with SMRTbell cleanup beads (PacBio, USA). Sequencing was carried out on a PacBio Revio system (PacBio, USA). Reads were processed with ccs (PacBio) using “--min-passes 3 --min-rq 0.99 --minLength 500,” and reads shorter than 2,000 bp were discarded. The resulting PacBio data were assembled with Hifiasm v0.12 (12), and a hybrid assembly with Illumina reads was created using Pilon v1.22 (13). Circularity was checked and confirmed using Circlator v. 1.5.5. Lastly, assembled sequences were annotated using the NCBI prokaryotic genome annotation pipeline (v6.9) (14).

**Fig 1:**
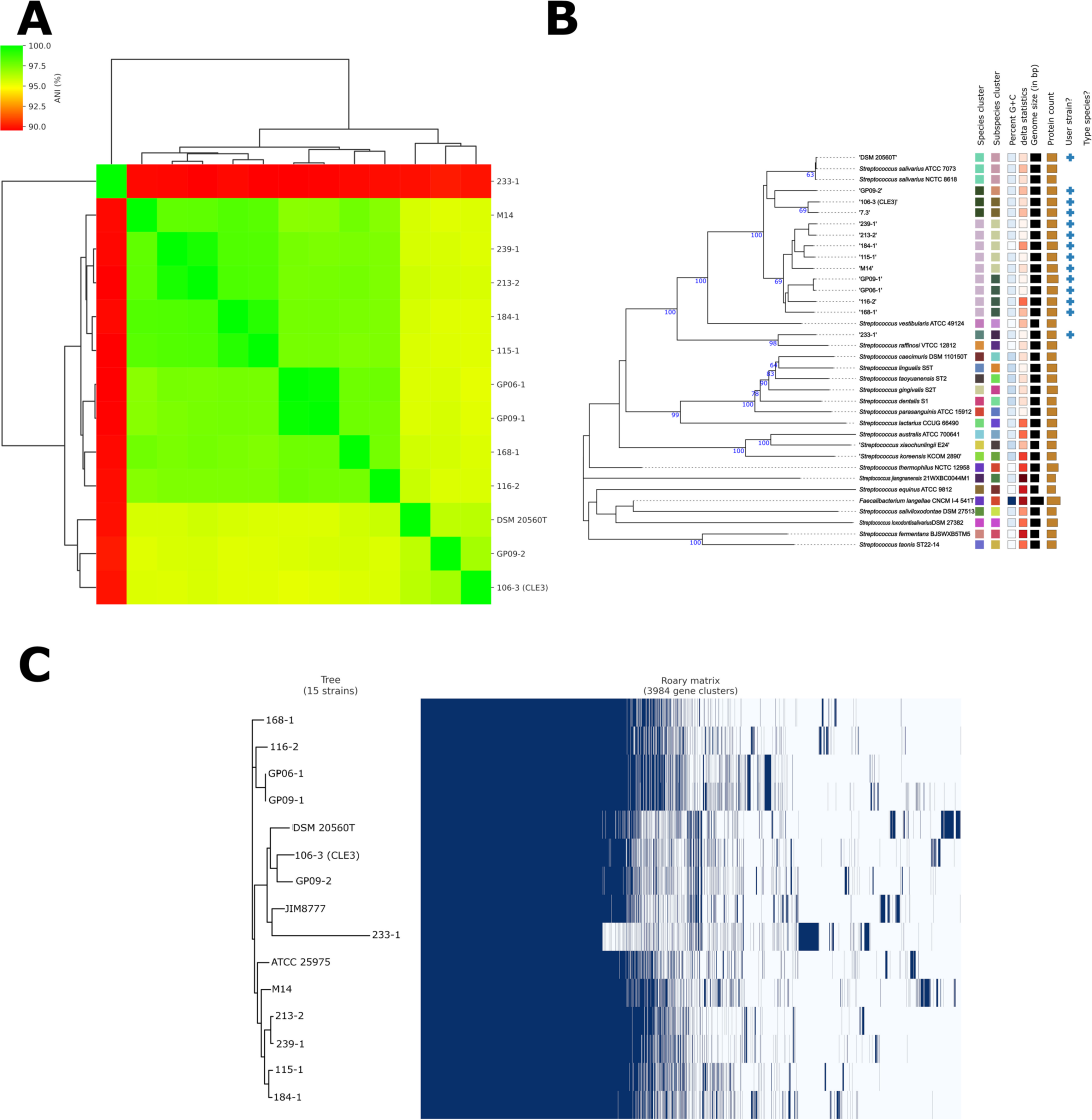
Genetic analysis confirming species and showing genetic diversity. (**A**) Average nucleotide index heatmap generated with ANIclustermap (15). A clustering of two major clades based on genome sequence similarity is visible. Additionally, strain 233-1’s ANI is significantly different from the rest of the strains indicated by the red color. (**B**) Phylogenetic tree based on whole-genome sequences generated using TYGS (17), showing 12 strains in a clade with *S. salivarius*, while 233-1 groups with *S. raffinosi*. (**C**) Whole-genome alignment of the pangenome using Roary (18) with 85% identity threshold and the 13 sequenced strains, as well as JIM8777 (accession number FR873482) and ATCC 25975 (accession number CP015283) as references. The blue color in the Roary matrix indicates the presence or absence of genes in the genome.

Key parameters of the sequencing results are summarized in Table 1. Additionally, to check for genetic diversity, ANIclustermap (15) was used to generate an average nucleotide index heatmap (Fig. 1A). Here, two major clades were identified, one containing the reference strain DSM 20560T, and strains GP09-2 and 106-3, the second clade including the remaining strains, except for strain 233-1, which, in further analysis, was deemed to be most closely related to the different, recently described species, *S. raffinosi* (16). The two clades, the species, and genetic distance were additionally confirmed using TYGS (17) and Roary (18) (Fig. 1B and C).

## Data Availability

The sequence data generated in this project have been deposited in the GenBank database. The data are available under the accession numbers referenced in Table 1 or under the BioProject accession number PRJNA1219549.
